# Reviews

**Published:** 1880-05

**Authors:** 


### REVIEWS.

---

**Hypodermic Injection of Morphia; its History, Advantages and Dangers.** By H. H. KANE, M. D. New York: Chas L. Birmingham & Co., publishers.

The thanks of the profession are due to Dr. Kane for this admirable book, which deserves a place in the library of every physician. The book records the experience of 360 physicians in answer to certain questions which were generally published in the medical journals. The opinions and cases thus collected furnish abundant food for thought, and the conclusions drawn therefrom, by the author of the book, are suggestive and highly valuable. In the chapter on deaths from the subcutaneous injection of morphia, 36 cases are reported. One correspondent (name not given) reports a death from the injection of 1-5 of a grain of morphia combined with 1-75 of a grain of atropia. In this number of the JOURNAL we publish a series of questions from Dr. Kane on hydrate of chloral. The admirable manner in which he has worked up the information obtained by his previously published questions, entitles him to the confidence of the profession. D.

---

**Skin Diseases; including their Definition, Symptoms, Diagnosis, Prognosis, Morbid Anatomy and Treatment, etc.** A Manual for Students and Practitioners. By MALCOLM MORRIS, Joint Lecturer on Dermatology at St Mary's Hospital Medical School, etc., with illustrations. Philadelphia: Henry C Lea. 1880. Buffalo: Theo. H. Butler.

This manual contains 316 pages, and is written in a plain and simple style, usually adopted by lecturers in presenting their subjects to medical classes. It has also the merit of conciseness, and presents much that is valuable for the student and general practitioner in this difficult and obscure class of diseases. Not unlike other works of this kind, the brevity with which many subjects of great importance are treated, deprives them of the clearness and accuracy of description, which tends to a superficial and imperfect study of medical subjects. The author however, intends the work to supplement, not to supplant, existing treatises on skin diseases, and with this end in view, he has rendered good service to the profession. L.

---

**Clinical Lectures on the Diseases of Women, delivered in St. Bartholomew's Hospital.** By J. MATHEWS DUNCAN, M. D., LL.D., F. R. S. E., etc. Philadelphia: Henry C. Lea. 1880.

This small volume of 175 pages comprises Dr. Duncan's clinical lectures, delivered in Saint Bartholomew, originally published in the English Medical Journals, and now reproduced in a separate form. The wide reputation of the author on all subjects connected with gynecology is an ample guaranty that valuable and well-digested matter is furnished to the profession in whatever issues from his pen. A critical examination of this work unfolds so much that is indispensable to those interested in this class of subjects, presented also with singular clearness and beauty, that we regard it as a valuable contribution to gynecological literature. The scope of the work does not enable the author to treat of many subjects, but his selection is just what the general practitioner requires for his daily work. L.

---

**A Manual of Auscultation and Percussion, embracing Physical Diagnosis of Diseases of the Lungs and Heart, and Throracic Aneurism** By AUSTIN FLINT, M. D. Second edition, revised Philadelphia: Henry C. Lea. 1880. Buffalo: Theo. H. Butler.

This work contains the substance of the lessons given in connection with practical instruction in auscultation and percussion to private classes, composed of medical students and practitioners. The author avoids all needless refinements, endeavors to consider the distinctive characters of the different physical signs by analysis, and lays stress upon the fact that knowledge of the significance of signs, rests solely on the constancy of their connection with the physical conditions which they represent. It is only necessary to state that this manual bears upon every page, the erudition, experience and skill of its accomplished author, and will be greeted by the profession as another of those valuable contributions to medical literature, which have placed Prof. Flint in the foremost position among medical writers. L.

---

**A Manual of Pathological Histology.** By V. CORNIL, Assistant Professor in the Faculty of Medicine, of Paris, and L. Ranvier, Professor in the College of France. Translated with notes and additions, by E. O. Shakespeare, A. M., M. D., Microscopist to the Philadelphia Hospital, and J. H. C. Simes, M. D., Demonstrator of Pathological Histology, &c., in the University of Pennsylvania. With 360 illustrations. Philadelphia: Henry C. Lea. Buffalo: Theodore Butler.

The original work of MM. Cornil and Ranvier has had a high reputation in France. The American translators have done their work so well that the American version is in every way superior, and we believe that it will be generally recognized as the best text book published in this country. The French work having been issued some years ago, is in some respects behind the present state of our knowledge; but in this edition we find that wherever the progress of the science has called for it, additions and changes have been made, with ability and excellent judgment, and the book is now a faithful exponent of the subject in its present state. The most extensive changes are in the sections on *sarcoma*, *carcinoma*, *tuberculosis*, and the classification of tumors.

The illustrations are numerous and well executed, and the mechanical part of the work is all that could be desired. D.

---

**On the Internal use of Water for the Sick and in Thirst.** A clinical lecture at the Pennsylvania Hospital, Oct 25, 1879. By J. FORSYTH MEIGS, M. D. Philadelphia: Lindsay & Blakiston. 1880.

The author quotes on the title page a trite old English proverb: "Drinking water neither makes a man sick, nor in debt, nor his wife a widow," and with so suggestive a text he presents a monograph replete with practical thoughts upon the use of water, as a hygienic and therapeutic agent. Its careful perusal by the profession will call to mind many plain indications for the use of water, rather than drugs, in the treatment of disease. The principles therein inculcated are well worth consideration by every medical man. L.

---

**The Student's Guide to Diseases of the Eye.** BY EDWARD NETTLESHIP, F. R. C. S. Philadelphia: Henry C. Lea.

Although several similar manuals are already before the profession, this one deserved more than passing notice. The writer has succeeded in condensing into a small space most of the leading facts of ophthalmology and presenting them in a clear, forcible manner.

The means of diagnosis are set forth in three short chapters, which, in general, give such directions for the examination of the external and internal portions of the eye, as to enable the student to see clearly the different features of a case, and to appreciate their significance. A good foundation having been thus laid, there follows the "Clinical Division," which naturally occupies most of the book.

In this, the diseases of the various structures are taken up in order, and treated in a simple and concise manner. No undue effort at originality is made, but well-established facts in diagnosis and treatment are so presented as to be easily remembered by the student, or referred to by the practitioner. The chapter which treats of "Diseases of the Eye in Relation to General Diseases," is a good epitome of this phase of the subject, and well worth reading.

The operations are, for the most part, grouped together in one chapter, and although this is in imitation of some of the most elaborate German works, it can hardly be considered the best arrangement. Moreover a few lithographic plates, representing diseases of the fundus, would add much to the value of such a hand-book without materially increasing its price, and a small collection of test-types, and one or two diagrams for the recognition of astigmatism, would also prove very convenient.

As a whole, however, we have here the most recent facts in ophthalmology set forth more clearly and in a smaller space than in any similar compendium now available to the English or American medical students. H.

---

**American Health Primer—Our Homes.** By Henry Hartshorne, A. M., M. D. Philadelphia: Presley Blackiston, 1012 Walnut St. 1880.

This little work is the ninth of the series, and contains practical and valuable hints and information in regard to the situation, construction, light, warmth, ventilation, water supply, drainage, disinfection of our homes. It is written in a popular style, suited to the general reader, and will be of essential service to the medical profession in disseminating facts on sanitary matters. L.

**Muscle-Beating; or Active and Passive Home Gymnastics, for Healthy and Unhealthy People.** By C. KLEMM, Manager of the Gymnastic Institution in Riga. With ten Illustrations. Price, 30 cents New York: M. L. Holbrook & Co

This work is a novelty and very suggestive, and will prove a valuable addition to the numerous modes of exercise, especially for chronic invalids and sedentary persons. L.

---

**Photographic Illustrations of Skin Diseases.** By GEORGE HENRY FOX, A. M., M. D. Complete in twelve parts; forty-eight colored plates, taken from life. Price $2.00. New York: E. B. Treat, 805 Broadway.

We have taken occasion before to refer in commendatory terms to the great service which Prof. Fox has performed in presenting to the profession these admirable illustrations of a class of diseases, concerning which there is a wide-spread ignorance existing because of a want of familiarity with their varied and multiform phases.

Parts 7, 8, 9, 10 recently received, more than justify all that has been previously written of these valuable works.

Part 7 treats of lupus vulgaris, lupus erythematosus, epithelioma superficiale and rodens, and epithelioma, and contains plates beautifully illustrating these diseases, which, for study, are quite equal to actual cases.

Part 8 treats of trichophytosis capitis and corporis, lichen planus and lichen ruber, with plates, taken from life, giving a wonderfully accurate idea of these diseases.

Part 9 treats of kerion, lepra maculosa, molluscum and erythema multiform.

In this number the plate illustrating erythema multiform is so life-like, that we doubt if any could ever mistake the disease, after studying this illustration with the accompanying text.

Part 10 treats of phtheiriasis capitis and corporis, scabies and porrigo pediculosis.

The work fulfills all the assurances given by the publisher for accuracy and completeness, and constitutes the best guide and help yet published in the study of dermatology. L.

